# GPCR-Gα13 Involvement in Mitochondrial Function, Oxidative Stress, and Prostate Cancer

**DOI:** 10.3390/ijms25137162

**Published:** 2024-06-28

**Authors:** Di Wu, Patrick J. Casey

**Affiliations:** 1 Programme in Cancer and Stem Cell Biology, Duke-NUS Medical School, 8 College Road, Singapore 169857, Singapore; di.wu@u.duke.nus.edu; 2 Department of Pharmacology and Cancer Biology, Duke University Medical Center, 308 Research Drive, Durham, NC 27710, USA

**Keywords:** G protein-coupled receptor, Gα13, Gα12, mitochondria, oxidative stress, antioxidant, superoxide dismutase 2, prostate cancer

## Abstract

Gα13 and Gα12, encoded by the *GNA13* and *GNA12* genes, respectively, are members of the G12 family of Gα proteins that, along with their associated Gβγ subunits, mediate signaling from specific G protein-coupled receptors (GPCRs). Advanced prostate cancers have increased expression of GPCRs such as CXC Motif Chemokine Receptor 4 (CXCR4), lysophosphatidic acid receptor (LPAR), and protease activated receptor 1 (PAR-1). These GPCRs signal through either the G12 family, or through Gα13 exclusively, often in addition to other G proteins. The effect of Gα13 can be distinct from that of Gα12, and the role of Gα13 in prostate cancer initiation and progression is largely unexplored. The oncogenic effect of Gα13 on cell migration and invasion in prostate cancer has been characterized, but little is known about other biological processes such as mitochondrial function and oxidative stress. Current knowledge on the link between Gα13 and oxidative stress is based on animal studies in which GPCR-Gα13 signaling decreased superoxide levels, and the overexpression of constitutively active Gα13 promoted antioxidant gene activation. In human samples, mitochondrial superoxide dismutase 2 (SOD2) correlates with prostate cancer risk and prognostic Gleason grade. However, overexpression of SOD2 in prostate cancer cells yielded conflicting results on cell growth and survival under basal versus oxidative stress conditions. Hence, it is necessary to explore the effect of Gα13 on prostate cancer tumorigenesis, as well as the effect of Gα13 on SOD2 in prostate cancer cell growth under oxidative stress conditions.

## 1. GPCR-Gα13 Signaling

### 1.1. Overview

G protein-coupled receptors (GPCRs) are seven-transmembrane receptors that form the largest family of transmembrane receptors, with over 800 in the human genome. GPCRs are involved in processes as diverse as embryogenesis, normal physiological functions, including metabolic and neuroendocrine control, as well as in the pathological processes of tumorigenesis and tumor progression [1]. As such, GPCRs govern a wide range of functions and processes, including sensory, neurotransmission, endocrine, exocrine, cell growth, and cell migration [2,3], several of which are also involved in malignancy. GPCRs are largely located at the plasma membrane and respond to diverse extracellular stimuli from both small molecule hormones and neurotransmitters, as well as peptides and even large protein signaling molecules [2]. More recently, it was recognized that GPCRs are also located at the membranes of intracellular compartments such as endoplasmic reticulum, Golgi, endosomes, cell division compartments, and inner and outer nuclear membranes [3,4]. In regard to cancer, ligand activation of overexpressed wild-type GPCRs, or constitutively active mutant GPCRs, can induce oncogenic transformation in vitro and in vivo [1,5,6,7,8]. In human samples, nearly 20% of cancers harbor GPCR mutations [9,10]. Furthermore, GPCR signaling drives cancer cell stemness, treatment resistance, and progression [11]. Hence, aberrant GPCR signaling likely contributes to tumorigenesis in many cell types and can affect cellular processes in different cellular compartments.

GPCRs are coupled to heterotrimeric G proteins, which consist of α, β, and γ subunits. There are 21 Gα proteins, 6 Gβ proteins, and 14 Gγ proteins [12,13,14]. Gα proteins are bound to guanosine diphosphate (GDP) in the inactive state and exchange for guanosine triphosphate (GTP) when activated by GPCR. This triggers the dissociation of Gα-GTP from Gβγ dimers, both of which can activate distinct signaling pathways. Once activated, GPCRs are often quickly desensitized via phosphorylation by GPCR kinases and coupling to β-arrestin or ubiquitination, then internalized by endocytosis, dephosphorylated, or deubiquitinated, and recycled to the membrane to couple with Gα-GDP and Gβγ [15]. This forms the classic cycle of GPCR activation.

**Figure 1:**
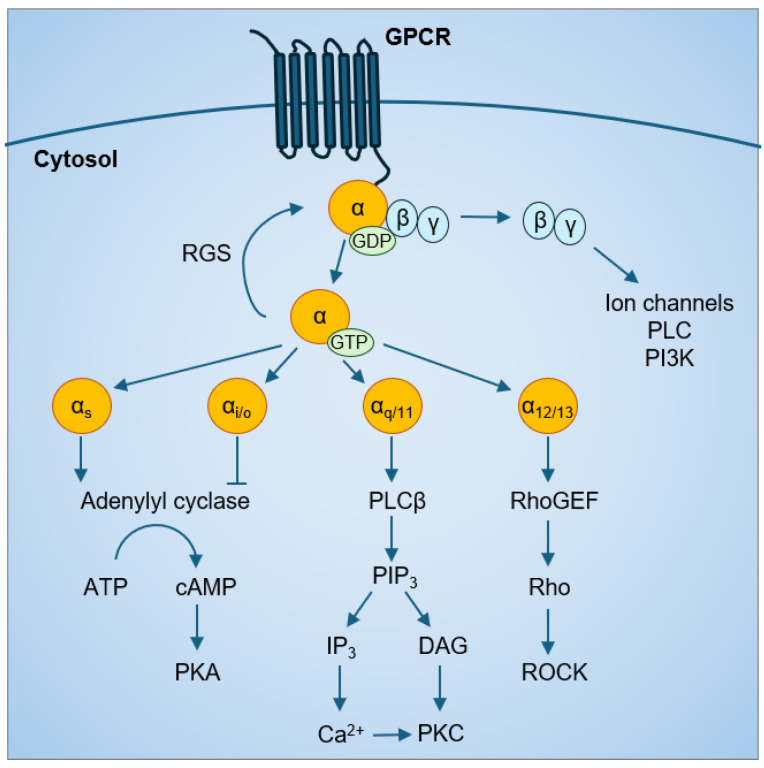
GPCR-Gα protein signaling.

Based on sequence homology and functional similarities, Gα proteins are categorized into four major families: Gαs, Gαi/o, Gαq/11, and Gα12/13. Each Gα family has preferential downstream effectors but can also activate multiple signaling cascades (Figure 1) Both the Gαs and Gαi family affect the cyclic adenosine monophosphate (cAMP) signal pathway through their effects on adenylyl cyclase; Gαs is stimulatory, while Gαi is inhibitory [16,17,18]. Gαq activates phospholipase Cβ and the phosphatidylinositol pathway to increase intracellular calcium levels [19]. Gα12/13 mainly activate the Rho family of GTPases (Rho) and the Rho-associated protein kinases (ROCK) pathways. Following Gα protein activation by a GPCR, the intrinsic GTPase activity of Gα proteins converts the bound GTP to GDP, which is the inactive Gα-GDP state. In addition, regulator of G protein signaling (RGS) proteins have GTPase activating protein (GAP) activity that can accelerate the intrinsic GTPase activity of Gα proteins [20], prevent Gα-GTP binding to effectors [21,22], and enhance Gα affinity for Gβγ binding [23,24]. This forms the classic cycle of Gα protein activation.

The Gα12/13 family consists of Gα12 and Gα13 proteins that are 67% homologous to each other [25]. Different from other families of Gα proteins, which share less than 45% identity with the Gα12/13 family, Gα12 and Gα13 are not targets of pertussis toxin or cholera toxin [26]. Activated Gα12/13 are capable of stimulating guanine nucleotide exchange factors (GEFs) for another class of GTPases, the Rho/Rac small GTPases [27]. For example, p115-RhoGEF [28,29], leukemia-related RhoGEF (LARG) [30,31,32], and PDZ, PSD-95/Dlg/ZO-1 homology-RhoGEF (PDZ-RhoGEF) [33,34] can activate the downstream Rho [35,36,37,38,39], cell division control protein 42 homolog (CDC42) [40], and Rac small GTPases [41]. Then, activated Rho-GTP binds to ROCK at its the carboxyl-terminus containing the Rho-binding domain. There are two ROCK isoforms, ROCK1 and ROCK2, and both bind to Gα12/13. Gα12/13 signaling via ROCK can increase phosphorylation of myosin phosphatase target subunit 1 (MYPT1), myosin light chain (MLC) [31,42] and the LIN-11, Isl-1, MEC-3 kinases (LIMK) [43] to regulate actin cytoskeletal remodeling, which, in turn, can activate the YAP/TAZ pathway [44,45].

Gα12/13 signaling can also regulate mitogen-activated protein kinase (MAPK) [40,46,47,48,49,50,51], phospholipases [37,51], protein kinases [52,53,54], Na^+^/H^+^ ion channels [40,48,54,55,56], and the Hippo signaling pathway [57]; whether all these require initial activation of Rho/Rac is not clear. These signaling pathways converge in the nucleus to regulate transcription factor activity and gene transcription. Gα12/13-regulated transcription factors include the nuclear factor kappa B (NF-κB) [53,58,59], activator protein 1 (AP-1) [41,60], serum response factor (SRF) [61,62], nuclear factor of activated T-cell c1 (NFATc1) [63,64], signal transducer and activator of transcription 3 (STAT3) [65], and yes-associated protein and the transcriptional coactivator with PDZ-binding motif (YAP/TAZ) [57,66].

Both in vitro models as well as results from knockout mice, both individually and in combination, have linked Gα12/13 signaling to numerous normal physiological processes, such as embryonic development and cell differentiation [63,67,68], cell metabolism, cell growth, actin cytoskeletal remodeling [35,69,70], cell migration [63], angiogenesis [71], platelet activation [72], osteogenesis [63,64,73], immune response [74,75], and neuronal responses [36,76,77]. Aberrant Gα12/13 signaling is also involved in oncogenic transformation [49,78,79], cancer cell growth [60], apoptosis [80,81,82], metastatic invasion [39,83,84], hypertension [31,85], inflammation, metabolic, fibrotic, and renal diseases [55].

Although Gα12 and Gα13 signaling appear to be very similar, their biochemistry and function are not completely overlapping [26]. Gα13 knockout is embryonic lethal in mice between embryonic day 9 and 10 due to angiogenic defects [86], whereas Gα12 knockout is viable without apparent abnormalities [87,88]. Moreover, Gα12 and Gα13 activate Na^+^/H^+^ exchanger via different signaling mechanisms [54] and have contrasting roles in osteogenesis [63,64,73]. In addition, Gα13, but not Gα12, enhances resistance against cytotoxic drugs and irradiation in primary head and neck squamous cell carcinomas [81]. It is purported that downstream effectors are able to discriminate Gα12 and Gα13, possibly due to the difference in abundance and cellular localization in addition to their sequence differences [26]. Gα12 is mainly located at the plasma membrane, while Gα13 appears to be located in the cytoplasm in multiple cell types [89]. Furthermore, some GPCRs selectively couple to either Gα12 or Gα13 [55,90,91]. In addition to the conventional model of GPCR-Gα13 signaling, Gα13 can also be activated independent of GPCR activation [92,93,94] or interact with non-GPCRs such as receptor tyrosine kinases (RTKs), smoothened [95], and integrins [96,97,98,99]. Hence, exploring the role of Gα13 protein will add to the current knowledge of the functions of Gα12 protein to increase the understanding of the biology of the Gα12/13 family.

### 1.2. Gα13 in Solid Cancers

Gα12 and Gα13 are also known as *gep* proto-oncogenes and are the only Gα proteins that induce oncogenic transformation when overexpressed, even without mutation [78,100,101]. Multiple studies have shown that Gα12/13 family can impact tumorigenesis, cancer cell migration and invasion, and drug resistance [102,103,104,105,106]. While the role of Gα13 in tumorigenesis and progression can be distinct from Gα12, it is relatively less studied, even though the oncogenic role of Gα13 was discovered several decades ago when overexpression of the *GNA13* cDNA in NIH3T3 fibroblasts was found to confer cell transformation [101]. Subsequent in vitro studies have mainly focused on the constitutively active Gα13 (Q226L) mutant, which does not exist in nature [49,79,101,107]. The only naturally occurring activating Gα13 mutation is at the arginine-200 site in bladder cancer [66], as well as inactivating mutations found in certain B cell lymphomas (a field of its own, and not covered here) [108,109]. In addition to being tumor-suppressive in lymphomas, Gα13 has also been reported to be tumor-suppressive in vitro in melanoma [110,111], glioma [112], and in genetically engineered KRas/Tp53 pancreatic cancer mouse models [113]. However, Gα13 is thought to be largely oncogenic in humans, where the most common alteration in Gα13 is *GNA13* gene amplification [114] or in enhanced activity of GPCRs coupled to Gα13, such as PAR-1 [115,116], CXCR4 [117], LPAR [118,119], thromboxane A2 receptor (TA2R) [9], and thyrotropin receptor (TSHR) [120]. This section will mainly focus on the role of Ga13 in solid cancers.

The oncogenic role of Gα13 is supported by patient data. The overall survival rate or progression-free survival rate is worse in those expressing higher levels Gα13 compared to those with lower levels of Gα13. Gα13 is a prognostic factor in gastric [121], hepatocellular [122], esophageal [123], and head and neck [81] cancers (Table 1). A meta-analysis of overall survival outcomes across 39 cancer types from ~18,000 human samples showed that *GNA13* gene expression is associated with poor prognosis in breast, lung, kidney, and ovarian cancers [124].

**Figure 2:**
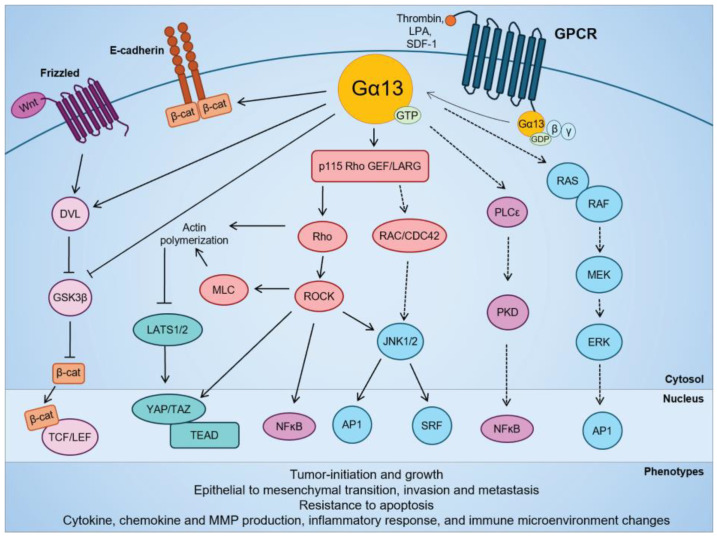
Signaling pathways regulated by Gα13 in cellular transformation and in solid cancers. Modified from Rasheed et al. (2022) [143].

The activation of Gα13 promotes in vitro cell proliferation and in vivo tumor growth in several cancer cell types such as head and neck squamous cell carcinoma [81], lung squamous cell carcinoma [125], small cell lung cancer [126], gastric cancer [121], and ovarian cancer [57] cells. The oncogenic transformation and mitogenic effects of Gα13 is at least partially mediated by RhoA-dependent activation of MAPK pathways (c-Jun, c-Jun N-terminal kinase (JNK), serum response factor), NF-κB and YAP/TAZ transcription factors [57,66,81,115,116,127,128] (Figure 2). Interestingly, overexpression of Gα13 did not promote cell proliferation in hepatocellular cancer cells [122], and expression of the constitutively active Gα13(Q226L) mutant in breast [83,129] and prostate [84,130] cancer cells had no effect on cell growth. However, limited studies have assessed the effect of wild-type Gα13 on anchorage-independent proliferation or tumor growth in mouse xenografts [81,83,125,131]. Hence, the oncogenic effect of Gα13 can be context-specific and cell type-dependent, and it is necessary to study the effect of wild-type Gα13 at endogenous levels in anchorage-independent growth models.

In vitro studies have revealed the role of Gα13 in other hallmarks of cancer. Gα13 is involved in cancer cell migration and invasion in solid cancers, such as prostate [130,132], breast [83,129], ovarian [57,131,133], lung [125], hepatocellular [122], pancreatic [134,135], and colorectal [136,137] cancers. Gα13 also protects against cell death induced by cytotoxic drugs or γ-irradiation in head and neck cancers [81], hepatocellular [138], and non-small cell lung cancer [139]. This enhanced resistance against cytotoxicity seems to be a function of Gα13, but not Gα12 [81]. In support of this, ROCK signaling also protects against apoptosis in prostate cancer [140], bladder cancer [141], and leukemia [142]. However, there are other hallmarks of cancer that are yet to be explored for Gα13, such as the regulation of metabolism.

### 1.3. Gα13 in Mitochondrial Function

There is limited evidence on the role of Gα13 on mitochondrial processes. Although Gα13 does not appear to be present in the mitochondria of cells [144], it has been reported to alter the expression of genes involved in mitochondrial biogenesis, oxidative phosphorylation and insulin insensitivity in osteoclasts [73], muscle [145], and liver [146], as seen in mice with tissue-specific deletion of *GNA13*. Moreover, Gα13 potentially mediates mitochondrial trafficking in neurons in *GNA12* and *GNA13* double-knockout HEK293 cells [147]. Additionally, several studies have shown that GPCR signaling regulates mitochondria morphology and cellular metabolism in normal cells [148,149,150,151]. Even though these studies indicate that Gα13 negatively regulates mitochondrial functions in non-cancerous cells, the role of Gα13 on the mitochondria in cancer cells and its effect on tumorigenesis is largely unstudied. Furthermore, GPCRs are localized and function on the mitochondrion [3,152], but none of these mitochondrially localized GPCRs have been shown to stimulate Gα13 proteins. In studies on human cancers, the GPCR succinate receptor 1 (SUCNR1/GPR91) inhibited mitochondrial respiration in gastric, lung, and pancreatic cancer cell lines that were addicted to glutamine [153]. In another study, the GPCR ligand lysophosphatidic acid (LPA) stimulated a transient increase in mitochondrial calcium ion uptake in colorectal cancer cells [154]. Finally, the GPCR ligand stromal cell-derived factor 1 (SDF-1), also known as CXC motif chemokine ligand 12 (CXCL12), induced mitochondrial superoxide dismutase 2 expression in human hepatocellular carcinoma cells [155]. Again, none of these plasma membrane GPCRs, even though capable of activating Gα13, have been yet shown to impact mitochondrial function by such activation.

## 2. Oxidative Stress in Cancer

### 2.1. Overview

Reactive oxygen species (ROS) include superoxide anion (O_2_^•−^), hydrogen peroxide (H_2_O_2_), hydroxyl radicals (HO^•^), and singlet oxygen (^1^O_2_). They are mainly generated intracellularly by electron leakage during mitochondrial oxidative phosphorylation in the electron transport chain (ETC), at the endoplasmic reticulum (ER) during unfolded protein response, and at the plasma and nuclear membranes by nicotinamide adenine dinucleotide phosphate oxidase (NADPH oxidase, NOX) [156]. The oxidizing effect of ROS creates oxidative damage to DNA, proteins, and lipids, and its accumulation eventually promotes tumorigenesis [156,157]. The strongest evidence for ROS-induced tumorigenesis is from superoxide scavenging by superoxide dismutase (SOD), the enzyme that catalyzes the transformation of superoxide to H_2_O_2_. Mice deficient in cytoplasmic Sod1 developed liver cancer [158], and mice with heterozygous deletion of mitochondrial Sod2 formed lymphoma and pituitary adenoma [159]. Mice deficient in peroxiredoxin-1, which reduces H_2_O_2_ to H_2_O, developed several types of malignancies [160]. In addition, mice deficient in antioxidant gene transcription factor nuclear factor erythroid 2-related factor 2 (Nrf2) demonstrated increased sensitivity of chemical carcinogenesis [161].

While many studies have focused on nuclear H_2_O_2_ that induces oxidative damage on genomic DNA, H_2_O_2_ is a relatively weak oxidant compared to superoxide [156]. In addition, mitochondria oxidative stress can directly lead to intrinsic apoptosis or other forms of cell death [162,163,164]. The major source of mitochondrial ROS generation is by ETC complex I and III [165], which are components of oxidative phosphorylation in energy metabolism. Complex I and III produce superoxide anions, which are catalyzed to H_2_O_2_ by SOD2 in the mitochondrial matrix and SOD1 in the intermembrane space. Then, H_2_O_2_ is catalyzed to H_2_O by catalase, glutathione peroxidase, and peroxiredoxin. Therefore, targeting the initial superoxide accumulation and oxidizing effects might be more effective than targeting H_2_O_2_. Moreover, anchorage-independent growth leads to metabolic reprogramming and increase in mitochondrial superoxide production [166,167,168], which could enhance the tumor cell inherent sensitivity to superoxide levels.

### 2.2. Gα13 in Oxidative Stress

**Figure 3:**
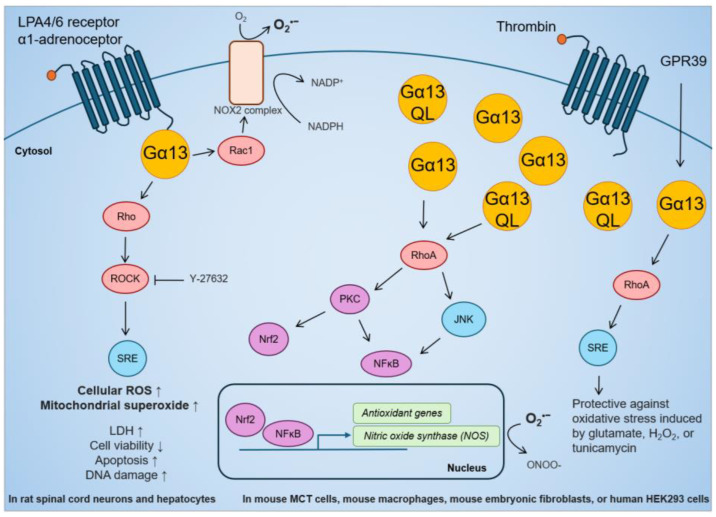
Effect of GPCR-Gα13 stimulation, overexpression of Gα13, or constitutively active Gα13(Q226L) mutant on cellular ROS, antioxidant gene activation, and resistance against oxidative stress in rat and mouse studies.

The role of Gα13 in regulating oxidative stress in human cancer is only now beginning to be studied. Current knowledge on the link between Gα13 and oxidative stress is mostly from mouse and rat studies (Figure 3). While it has been reported that GPCR activation of Gα13 can increase oxidative stress, most studies have reached the opposite conclusion. In regard to the first, LPA stimulation in rat spinal cord neurons increased ROS detected by 2′,7′–dichlorofluorescin diacetate (DCFDA) and MitoSOX Red Staining, which was suppressed by ROCK inhibition [169]. This is supported by a study in rat hepatocytes in which stimulation of the GPCR α1-adrenoceptor activated the NADPH oxidase 2 (NOX2) complex via Gα13-Rac1 [170]. NOX2 transports electrons from NADPH to O_2_ and generates superoxide, which is rapidly converted into H_2_O_2_ [170]. In terms of the second, stable overexpression of Gα13 or Gα13(Q226L) in the mouse renal proximal tubule MCT cell line increased inducible nitric oxide synthase (iNOS) activity through mRNA transcription [171]. In mouse macrophage Raw264.7 cells, iNOS activity was induced by thrombin stimulation and Gα13(Q226L) expression [172]. iNOS catalyzes the production of nitric oxide (NO) that can react with superoxide to form peroxynitrite (ONOO^-^), which is a strong reactive nitrogen species (RNS) that has been shown to promote immune evasion in cancer cells [173]. The scope of this review does not further cover the role of Gα13 in RNS or nitrosative stress. Moreover, in mouse hippocampal neuronal HT-22 cells and human HEK293 cells, the overexpression of the GPCR GPR39 confers protection via the Gα13-RhoA-serum response element (SRE) pathway against oxidative stress induced by glutamate, H_2_O_2_, or tunicamycin [174]. Finally, in mouse embryonic fibroblasts, the expression of constitutively active Gα13(Q226L) mutant promoted Nrf2 activity and antioxidant gene transcription via Rho-protein kinase C-delta (PKCδ)-Nrf2 pathway [175]. Overall, Gα13 is clearly capable of inducing expression of antioxidant genes to suppress oxidative stress. However, the effect of Gα13 on oxidative stress in cancer biology needs to be further explored (see sections below).

## 3. Prostate Cancer

### 3.1. Overview

The prostate is an exocrine gland important for male reproduction that responds to the male hormones testosterone and dihydrotestosterone. Hence, androgen deprivation therapy (ADT) is the mainstay of prostate cancer treatment [176,177]. Despite being the second-most commonly diagnosed cancer in males [178,179], most prostate cancers are indolent and have low aggressiveness, with an overall five-year survival of 83–97% [180,181]. However, most ADT-responsive prostate cancers eventually become ADT-resistant, also known as castration-resistant prostate cancer (CRPC), which drastically increases morbidity and mortality with a median survival of 13–30 months [182]. Moreover, aggressive forms of prostate cancer and CRPC are more fatal in men under the age of 50 [183,184,185].

Although there is a strong genetic component to primary prostate cancer, no single driver mutation is solely responsible for tumorigenesis or aggressiveness [186]. In metastatic CRPC, the most frequently altered genes include the androgen receptor gene *AR* (62.7%), the erythroblast transformation specific (ETS) family (56.7%), tumor protein P53 gene *TP53* (53.3%), and phosphatase and tensin homolog gene *PTEN* (40.7%) [186,187,188]. The AR pathway is activated in all metastatic, androgen-independent CRPC through AR gene amplification, constitutively active mutations or overexpression, as well as mutations in AR coactivators [186,188]. In addition, the protein kinase B (AKT), MAPK and retinoblastoma (Rb) signaling pathways are altered with higher frequencies in metastases compared to primary tumors [188,189]. Crosstalk between these pathways and AR enhances prostate cancer tumor growth [190,191]. AR stimulation activates the Src-rapidly accelerated fibrosarcoma (RAF)-MAPK kinase (MEK) pathway, resulting in MAPK activation [192,193,194,195], while ADT-resistance activates the phosphatidylinositol-3 kinase (PI3K)-AKT-mammalian target of rapamycin (mTOR) pathway, leading to mitogenic growth [196,197,198,199,200,201]. AR signaling also phosphorylates Rb protein controlling cell cycle G1 to S phase progression [202,203,204], and dysfunctional Rb protein increases AR target gene expression [205,206].

Several clinical trials have targeted the aforementioned upregulated signaling pathways, either individually or synergistically with ADT. However, these new therapies have made only slight improvement in prostate-specific antigen (PSA) levels or progression free survival. For example, a phase III trial (IPATential150) evaluating the combination therapy of pan-AKT inhibitor ipatasertib and androgen biosynthesis inhibitor abiraterone found only slight improvement in median radiographic progression free survival (rPFS) of metastatic CRPC patients with PTEN deficiency (18.5 months versus 16.5 months; hazard ratio 0.77; *p* = 0.0335) [207,208]. In a phase II trial, PI3K/mTOR inhibitor samotolisib + AR inhibitor enzalutamide improved median rPFS than placebo + enzalutamide (10.2 vs. 5.5 months; *p* = 0.03) [209]. Another phase II trial on PI3K inhibitor buparlisib + enzalutamide improved median PFS compared to buparlisib alone (3.5 vs. 1.9 months) [210]. The phase III READY trial evaluating Src inhibitor dasatinib + anti-mitotic docetaxel versus docetaxel alone had no significant improvement in PSA levels or PFS [211]. Overall, these pathway inhibitors did not demonstrate significant activity in metastatic CRPC, suggesting that they are insufficient to reverse CRPC progression. Hence, exploring new biological mechanisms in prostate cancer is needed.

### 3.2. Gα13 in Prostate Cancers

Advanced prostate cancers exhibit increased expression of GPCRs such as CXCR4 [117], LPAR1-3 [118,119,212], and PAR-1 [213]. These GPCRs signal through either the Gα12/13 family, or through Gα13 exclusively [55,91]. In addition, stimulation with GPCR ligands such as LPA [214,215,216,217] and thrombin [213] can promote cell growth in both androgen-responsive and androgen-independent prostate cancer cell lines [218,219,220,221,222,223,224,225,226,227,228,229,230]. Moreover, exome sequencing data sets show that the *GNA13* gene is amplified in prostate cancer samples at a level of 4% (21/492) in primary adenocarcinoma in The Cancer Genome Atlas (TCGA) and at 28% (123/444) in metastatic CRPC in the SU2C/PCF data set [231,232,233]. *GNA13* mRNA levels also positively and significantly correlated to prognostic Gleason scores in the TCGA and SU2C/PCF data sets. Although Gα13 has not been directly linked to AR signaling, the stimulation of RhoA leads to transcriptional activation of AR [234]. Activation of AR is linked to androgen-independence and aggressive phenotypes. Although there is no available data on the immunohistochemistry staining of Gα13 in prostate cancer samples, the protein expression of Gα13 in prostate cancer cell lines correlates with androgen-independence and aggressiveness. Gα13 protein expression in the more tumorigenic, invasive, androgen-independent PC3 and DU145 cells is significantly higher than in the less tumorigenic, non-invasive, androgen-responsive LNCaP cell line [84,130,132]. Hence, Gα13 may play a significant role in prostate cancer biology.

**Figure 4:**
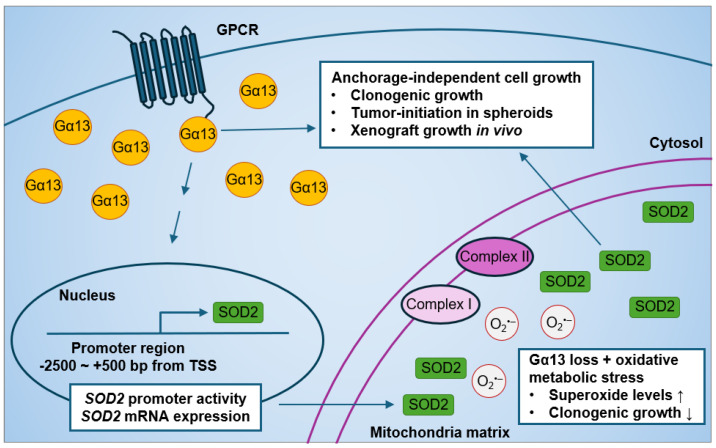
Effect of modulating Gα13 on mitochondrial SOD2 expression and anchorage-independent cell growth in human prostate cancer cells.

Current evidence on the effect of Gα13 on prostate cancer cell growth is limited to monolayer proliferation. Forced expression of an activated Gα13(Q226L) mutant has no effect on monolayer cell growth as assessed by thymidine incorporation assays after 12 h serum stimulation in both PC3 and Du145 cells [84]. Although the expression of p115-RGS (a dominant-negative inhibitor of both Gα12 and Gα13) in PC3 and Du145 cells suppressed RhoA activation by thrombin stimulation [84], its expression had no effect on short-term monolayer cell growth in PC3 and Du145 cells [84]. Stable expression of p115-RGS in PC3 cells also had no significant change in soft agar colony formation in soft agar or in xenograft tumors [84]. However, it was not known until recently whether silencing endogenous Gα13 rather than overexpressing the constitutively active Gα13(Q226L) mutant (that is not naturally occurring) affects prostate cancer anchorage-independent cell growth. In our recent study, we showed that the knockdown of *GNA13* in the high-Gα13-expressing PC3 cells reduced anchorage-independent cell growth in in vitro and in vivo models (Figure 4) [233]. Gα13 loss in PC3 cells suppressed soft agar colony formation, spheroid formation upon serial re-plating, and xenograft tumor growth. Likewise, the overexpression of Gα13 in the low-Gα13-expressing LNCaP cells promoted anchorage-independent cell growth. Hence, modulating wild-type Gα13 expression at endogenous levels in prostate cancer cells affects anchorage-independent cell growth in vitro and tumor growth in mouse xenografts.

The most well-characterized oncogenic effect of Gα12/13 in prostate cancer is cell migration and invasion. Modulation of Gα13 positively regulated RhoA activation in the prostate cancer cell lines PC3, Du145, and LNCaP [59,84,130,132]. The activated Gα13(Q226L) mutant promoted cell invasion in both PC3 and Du145 cells [84], whereas transient knockdown of *GNA13* in PC3 cells suppressed cell migration and invasion [130,132]. Overexpression of Gα13 in LNCaP cells increased invasion to CXCR4 ligand SDF-1/CXCL12 as chemoattractant [130]. In addition, Gα13 promoted Rho-dependent expression of chemokine CXCL5 via the NFκB complex [59]. Hence, it is worthwhile to explore other oncogenic biological processes in which Gα13 may impact.

Interestingly, we recently found a novel role of Gα13 in mitochondrial oxidative metabolic stress response (Figure 4) [233]. The effect of Gα13 loss on oxidative stress was more evident when cells were cultured in glucose-free media supplemented with non-glycolytic metabolites, such as galactose, succinate, glutamate, and malate, which, in turn, increased oxidative metabolic stress in the mitochondria. This effect of Gα13 on superoxide levels was specific to the mitochondria, as little to no change in cytosolic superoxide levels were detected in PC3 cells. Phenotypically, increased oxidative metabolic stress induced by non-glycolytic metabolites had no significant effect on colony formation in PC3 control cells. However, *GNA13* knockdown resulted in decreased soft agar colony formation when cells were cultured in galactose, succinate, glutamate, and malate as compared to glucose culture. These results suggest that Gα13 loss increases sensitivity to oxidative metabolic stress-induced non-glycolytic metabolites in PC3 cells.

### 3.3. Oxidative Stress in Prostate Cancer

Oxidative stress plays an important role in prostate cancer initiation and progression. The expression of antioxidant enzymes SOD1, SOD2, and catalase are lower in prostate cancer than in normal tissue [235]. Oxidative stress also crosstalks with AR signaling and is involved in the development of androgen-independent CRPC [236,237,238]. Androgen deprivation and silencing of AR increases intracellular ROS, which, in turn, promotes AR expression and signaling resulting in androgen-independence [238]. In patients, prostate cancer tissue specimens from patients who have received ADT had higher lipid peroxidation oxidative damage compared to those from patients who have not received ADT [239]. On the other hand, it seems that after prostate cancer cells become androgen-independent, the increased expression of antioxidant enzymes lowers intracellular ROS. Proteomics analysis of androgen-independent versus androgen-dependent LNCaP cells showed a higher expression of antioxidant enzymes such as antioxidant protein 2, SOD1, thioredoxin peroxidase, and peroxiredoxin-2 [240,241]. Moreover, androgen-resistant 22RV1 cells had lower intracellular H_2_O_2_ levels compared to control cells both before and after irradiation [242]. In addition, polymorphisms in the antioxidant *SOD2*, glutathione S-transferase mu 1 and 3 (*GSTM1* and *GSTM3*), glutathione S-transferase pi 1 (*GSTP1*), catalase (*CAT*), and paraoxonase 1 (*PON1*) genes have been associated with increased susceptibility to prostate cancer or increased risk of progression to metastatic CRPC [243,244]. Although a link between ROS in the form of H_2_O_2_ is linked to prostate cancer progression, less is known about the effect of ROS in the form of superoxide and of SOD enzymes on prostate cancer.

Mitochondrial SOD2 is associated with prostate cancer risk, whereas no significant association was detected for cytoplasmic/nuclear SOD1 or extracellular SOD3 [245]. In patient samples, SOD2 protein levels were significantly increased in tumor (Gleason 3–9) compared to hyperplasic or control tissues [246] and correlated with prostate cancer patient Gleason scores [247]. Control sample and low-grade tumor (Gleason 5) had much lower SOD2 levels than medium-grade tumors (Gleason 7), and high-grade tumors (Gleason 8) had even higher SOD2 protein levels. When androgen-responsive LNCaP cells were stimulated to neuroendocrine differentiation, the process also increased SOD2 protein levels [247]. Meta-analyses and other studies have concluded that *SOD2* (rs4880) polymorphism increases prostate cancer risk and aggressiveness [244,248,249,250]. Moreover, men homozygous for *SOD2* (rs4880) have increased odds for high-grade tumors [251,252]. However, homozygous *SOD2* (rs4880) was not predictive of prostate cancer recurrence [253] or overall survival [248,254] after radical prostatectomy. In addition, intratumoral SOD2 levels should be distinguished from serum SOD2 levels [255]. Given the relevance of SOD2 in prostate cancer patients, the role of mitochondrial SOD2 in prostate cancer tumorigenesis needs to be explored.

In addition to the roles of Gα13 in anchorage-independent cell growth and mitochondrial oxidative metabolic stress response in prostate cancer cells, we recently also found that modulating Gα13 levels impacted mitochondria SOD2 expression (Figure 4) [233]. Of note, both PC3 and LNCaP harbor the *SOD2* (rs4880) polymorphism [256]. First, we found strong positive correlations between *GNA13* and *SOD2* mRNA expression in The Cancer Genome Atlas database. This led to the finding that Gα13 lower-expressing LNCaP cells have lower SOD2 protein levels compared to Gα13 higher-expressing PC3 cells, which is consistent with previous studies [256,257]. Most importantly, we showed that knockdown of *GNA13* in PC3 cells reduced SOD2 protein expression in the mitochondria, as well as SOD2 mRNA expression and promoter activity. Likewise, the overexpression of Gα13 in LNCaP cells increased mitochondrial SOD2 expression. In contrast, modulating Gα13 in prostate cancer cells had no impact on SOD1 expression. Hence, it seems clear that Gα13 regulates SOD2 expression in prostate cancer cells.

**Figure 5:**
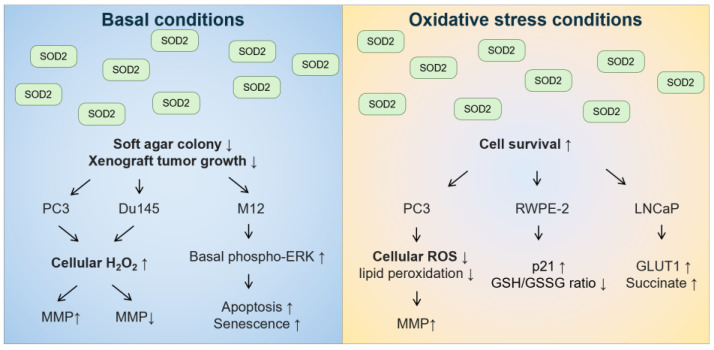
The effect of overexpressing SOD2 on anchorage-independent cell growth and cell survival under basal and oxidative stress conditions in human prostate cancer cell lines.

With regard to role of the mitochondrial SOD2 in prostate cancer tumorigenesis, the in vitro data are conflicting (Figure 5). Under basal conditions, the overexpression of wild-type SOD2 suppressed soft agar colony formation or xenograft tumor growth in androgen-independent PC3 [258] and Du145 cells [259], as well as in androgen-responsive M12 cells [260]. In these cell lines, overexpression of SOD2 or treatment with SOD2-mimetic resulted in changes in cell cycle with a delay in the G1 phase or a decrease in the G2+M phase [258,260,261]. Although the decreased growth rate was not due to necrosis, apoptosis, or senescence in PC3 cells [258], increased apoptosis and senescence were observed in M12 cells overexpressing SOD2 [260]. M12 cells overexpressing SOD2 also had increased basal levels of phosphorylated extracellular signal-regulated kinase (ERK) [260]. In both PC3 and Du145 cells, overexpression of wild-type SOD2 increased H_2_O_2_ levels [258,259]. Differences between PC3 and Du145 cells included that overexpression of SOD2 in the mitochondria increased mitochondrial membrane potential in PC3 cells [258,262] but decreased mitochondrial membrane potential in Du145 cells [259]. In contrast, under oxidative stress conditions, such as hyperthermia, PC3 cells with overexpression of SOD2 had better survival than control cells [262]. In addition, androgen-responsive RWPE-2 cells overexpressing wild-type SOD2 were less sensitive to high levels of sodium selenite that generate superoxide, but not to glutathione-depleting agent buthionine sulfoximine [263]. Moreover, androgen-responsive LNCaP cells overexpressing wild-type SOD2 were more resistant to glucose deprivation-induced cell death, whereby glucose deprivation can increase mitochondrial superoxide levels [264]. Hence, these data suggest that the effect of SOD2 on prostate cancer tumorigenesis should be distinguished between basal conditions and oxidative stress conditions, especially for conditions that increase superoxide stress.

As mentioned above, the knockdown of *GNA13* in PC3 cells reduced soft agar colony formation, suppressed mitochondrial SOD2 expression, and increased mitochondrial superoxide levels (Figure 4) [233]. The phenotypes displayed by SOD2 loss induced by *GNA13* knockdown were more evident under oxidative stress conditions. For example, the increase in mitochondrial superoxide levels induced by *GNA13* knockdown was more pronounced when cells were cultured in oxidative metabolic stress conditions (i.e., non-glycolytic metabolites). Moreover, mitochondrial oxidative metabolic stress further suppressed in colony formation that was already suppressed by Gα13 loss, and, in turn, SOD2 loss, in PC3 cells. Our data further validate that Gα13 and mitochondrial SOD2 promote cell survival under oxidative stress conditions in prostate cancer cells.

Many studies on antioxidants and prostate cancer have focused on suppressing ROS to, in turn, suppress AR expression and signaling. Natural antioxidants such as carotenoids can suppress prostate-specific antigen levels [265,266], and tocopherols improved overall prostate cancer survival in randomized, controlled clinical trials [267]. In in vitro studies, the antioxidant N-acetyl cysteine reduced AR expression, and along with ADT, it decreased xenograft tumor growth in androgen-responsive LNCaP and 22Rv1 cells [239]. SOD mimetics such as 4-hydroxy-2,2,6,6-tetramethylpiperidin-1-oxyl (TEMPOL), manganese(III) tetrakis(4-benzoic acid)porphyrin (MnTBAP) and manganese(III) tetrakis(1-methyl-4-pyridyl)porphyrin (MnTMPyP) reduced AR expression and decreased cell viability in androgen-responsive LNCaP, CWR22Rv1, and LAPC-4AD cells [268]. However, TEMPOL had little effect on androgen-independent PC3 cells [268]. Hence, it is necessary to further explore the effect of SOD2 in androgen-independent prostate cancer cells and to describe the effect of silencing SOD2, in contrast to overexpression, on prostate cancer growth.

It now seems clear that loss of Gα13 suppresses anchorage-independent cell growth in multiple prostate cancer cell lines. In this regard, in PC3 cells in which *GNA13* was silenced, the effect of Gα13 loss on suppression of anchorage-independent cell growth was rescued by overexpression of SOD2 in a fashion dependent on the catalytic activity of SOD2, since colony formation was not rescued when a catalytically inactive SOD2(Q167A) mutant was expressed. In support of this, anchorage-independent cell growth was attenuated upon SOD2 knockdown in Gα13-overexpressed LNCaP cells. Furthermore, at the time of tumor injection in the xenograft mouse model, PC3 cells in which *GNA13* was knocked down had approximately 40% lower SOD2 protein levels compared to control cells; this corresponded to a 40% reduction in xenograft tumor growth in *GNA13* knockdown cells compared to control cells. Overall, these studies highlight a novel biological route of Gα13-mediated anchorage-independent growth and response to oxidative metabolic stress through regulation of SOD2 expression in prostate cancer cells.

## 4. Conclusions

Prostate cancers harbor aberrant GPCR-Gα13 signaling, which has been linked to oncogenic transformation, cancer cell growth, migration, and invasion. Modulating wild-type Gα13 expression at endogenous levels in prostate cancer cells affects anchorage-independent cell growth in vitro and tumor growth in vivo. Furthermore, a novel role of Gα13 was recently found in mitochondrial oxidative metabolic stress response. Although a link between Gα13 and oxidative stress has been shown in animal studies, the data in human biology was lacking until now. While ROS in the form of H_2_O_2_ has been linked to prostate cancer progression, much less is known about the effect of ROS in the form of superoxide and of SOD enzymes on prostate cancer. Now that Gα13 has been implicated in mitochondrial SOD2 expression in prostate cancer cells, which has been shown to correlate with prostate cancer risk and prognostic Gleason score, it is clear that the role of Gα13 and mitochondrial SOD2 in prostate cancer tumorigenesis needs to be further explored.

## Figures and Tables

**Table 1:** Prognosis of patients with tumors expressing Gα13 protein at higher versus lower levels, as assessed by immunohistochemistry staining in tumor samples.

Cancer Type	No. of Patients Gα13 High/Low	Overall Survival	Disease-Free Survival
Head and neck squamous cell [81]	85/70	NA	Distant metastases-free survival (log-rank *p* = 0.032) Loco-regional recurrence (log-rank *p* > 0.05).
Gastric [121]	Training: 93/140 Validation: 90/103	Training: Log-rank *p* < 0.001; HR: 8.244; 95% CI: 2.495–9.510, *p* < 0.001. Validation: Log-rank *p* < 0.001; HR: 3.135, 95% CI: 1.819–5.401, *p* < 0.001.	NA
Hepatocellular [122]	148/98	Median 39.1 vs. 48.9 months; log-rank *p* = 0.003. HR: 1.558, 95% CI: 1.096–2.215, *p* = 0.014.	Log-rank *p* = 0.001. HR: 1.564, 95% CI:1.148–2.132, *p* = 0.005.
Esophageal [123]	85/88	Median 30.3 vs.54.9 months; log-rank *p* < 0.001). HR: 2.396, 95% CI: 1.540–3.726, *p* < 0.001.	Median 16.4 vs.53.3 months; log-rank *p* < 0.001. HR: 2.276, 95% CI: 1.441–3.594, *p* < 0.001.

Abbreviations: HR, hazard ratio by multivariable analysis; 95% CI, 95% confidence interval; NA, not available.
